# Should general practitioner access to breast imaging and 2WW co-exist?

**DOI:** 10.1186/bcr2988

**Published:** 2011-11-04

**Authors:** ME Fletcher, N Sharma, BG Dall

**Affiliations:** 1 Leeds Teaching Hospital NHS Trust, Leeds, UK

## Introduction

The Leeds breast service treats over 500 breast cancers a year. The general practitioners (GPs) have always had access via the one-stop breast clinic or direct access to the imaging department. Any positive imaging findings are actioned within imaging and the patients are referred to the clinic for results. The GP is informed. With the introduction of 2WW it is appropriate to re-audit this practise.

## Methods

This is a retrospective audit performed over a 6-month period using the radiology database (CRIS) and the clinical database (PPM). The data collected included the number of GP referrals, the imaging performed, the number of biopsies and the number of referrals to the breast clinic. The timeline for each patient was recorded and the costs involved where compared with referral to the one-stop clinic.

## Results

A total of 592 patients were referred direct to imaging from 1 July 2010 to 31 December 2010. The GP referrals were seen within an average of 23 days (3 to 47 days). A total of 223 patients had mammography and ultrasound, 165 had mammography only and 204 had ultrasound only. Twenty-six patients had biopsies; 45% were performed at the same attendance. Nine cancers were diagnosed. Forty-one patients were referred to the one-stop clinic. The radiology department received payment per procedure compared with a standard percentage per patient.

## Conclusion

The one-stop clinic with triple assessment is the gold standard. GP direct access still safely reduces the burden of a 2-week wait provided robust pathways are in place to ensure prompt biopsy and appropriate referral to the breast clinic.

